# Strong foraging preferences for *Ribes alpinum* (Saxifragales: Grossulariaceae) in the polyphagous caterpillars of Buff‐tip moth *Phalera bucephala* (Lepidoptera: Notodontidae)

**DOI:** 10.1002/ece3.6981

**Published:** 2020-11-11

**Authors:** Juliano Morimoto, Zuzanna Pietras

**Affiliations:** ^1^ School of Biological Sciences University of Aberdeen Aberdeen UK; ^2^ Department of Physics, Chemistry and Biology (IFM) Linköping University Linköping Sweden

**Keywords:** diet breadth, ecological specialization, niche, polyphagy, range shift

## Abstract

1. Herbivorous insects such as butterflies and moths are essential to natural and agricultural systems due to pollination and pest outbreaks. However, our knowledge of butterflies' and moths' nutrition is fragmented and limited to few common, charismatic, or problematic species.
2. This gap precludes our complete understanding of herbivorous insects' natural history, physiological and behavioral adaptations that drive how species interact with their environment, the consequences of habitat fragmentation and climate change to invertebrate biodiversity, and pest outbreak dynamics.
3. Here, we first report a population of the Buff‐tip moth *Phalera bucephala* (Lepidoptera: Notodontidae) feeding on a previously unknown family of host plants, the mountain currant *Ribes alpinum* (Saxifragales: Grossulariaceae). This is the first report of a Notodontid moth feeding on Grossulariaceae hosts.
4. Using no‐choice and choice assays, we showed that *P. bucephala* has strong foraging preferences for a previously unknown hosts, the *R. alpinum* but also, although to a smaller extent, *R. uva‐crispa* compared with a previously known host (the Norway maple *Acer* sp.).
5. These findings demonstrate that *P. bucephala* feed on—and show strong preference for Grossulariaceae host plants, indicating flexible physiological mechanisms to accommodate hosts plants from various families. This makes this species a potential model organism to study the behavioral and physiological mechanisms underpinning insect–plant interactions and diet breadth evolution.
6. We discuss the broad ecological implications of these observations to the biology of the species, the potential negative effects of interspecific competition with endemic specialist moths, and highlight questions for future research.

## Graphical abstract

Herbivorous insects are essential to natural and agricultural systems due to pollination and pest outbreaks, but our understanding of their natural history remains limited. We describe, for the first time, *Phalera bucephala* caterpillars feeding on—and strongly preferring—a previously unknown family of currants and gooseberry plants. This is the first report of a Notodontid moth feeding on Grossulariaceae hosts. Our observation has broad implications to the ecology the species and interspecific competition with endemic moth specialists.

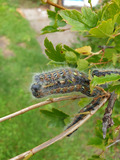

## 1 INTRODUCTION

Herbivorous insects display a wide variety of nutritional strategies in relation to diet, ranging from strict specialism (e.g., the Large Blue *Phengaris arion*, *Drosophila seichellia*) to broad generalism (e.g., *Lymantria dispar*, *D. suzukii*) (Forister et al., 2015). Diet breadth influences physiological, morphological, and behavioral adaptations that shape the evolutionary trajectory of populations (Deane Bowers & Puttick, 1988; Poisot et al., 2011; Roughgarden, 1972). A complete understanding of the diet breadth of a species provides fundamental knowledge about species' ecology, which can be useful for modeling species distribution and inform strategies for control of pest species (Clarke et al., 2011; Jaenike, 1990). Importantly, with the current recognition of the worldwide decline of insect species (Conrad et al., 2006; Didham et al., 2020; Saunders et al., 2020), particularly specialists (i.e., “functional homogenisation”, Clavel et al., 2011), there is an unprecedented urgency for documenting suitable host plants of threatened species as well as species that can become/are pests. With this knowledge, it is possible to incorporate natural history into eco‐evolutionary studies which will allow for informed decisions aimed to protect species in decline while mitigating negative effects of invasive or competitively superior species in a given ecosystem (Paine & Millar, 2002; Travis, 2020).

**FIGURE 1:**
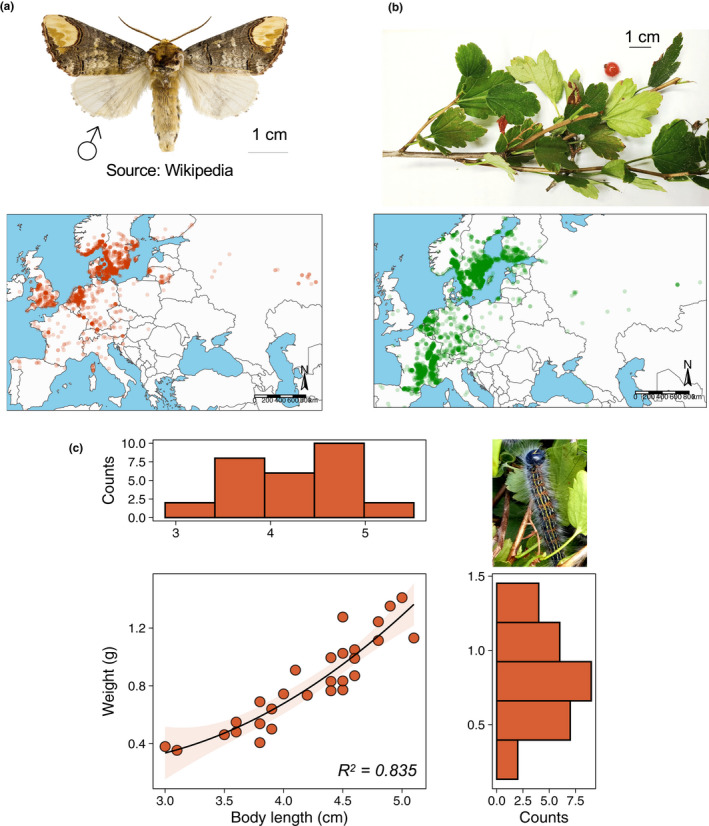
Natural history report of a previously undescribed host plant, the alpine currant, for the Buff‐tip moth. (a) Adult male *P. bucephala* (image from the public domain) and recorded observations (*N* = 5,000) of *P. bucephala*. (b) *R. alpinum* specimen from the collection site and recorded observations (*N* = 5,000) of *R. alpinum* in Europe. Data queried from the GBIF database on the July 27, 2020. (c) Morphometrics of third instars *P. bucephala* developing in *R. alpinum*. Top‐left panel: histogram of caterpillars' body length (in cm) in the sample. *Top‐right panel*: *P. bucephala* specimen feeding in *R. alpinum*. Note the characteristic “V” yellow mark in the caterpillars' head. *Bottom right panel*: histogram of caterpillars' body weight (in g) in the sample. *Main panel*: Relationship between caterpillars' body length and weight. Model fit obtained with a polynomial regression of degree = 2. Equation: Weight*_Ribes_* = (Length*_Ribes_*)^2^ × 0.134 − Length*_Ribes_* × 0.596 + 0.917

Here, we report a natural history observation of the Buff‐tip moth *Phalera bucephala* (Lepidoptera: Notodontidae) (Figure 1a) utilizing a previously unknown host plant, the alpine currant *Ribes alpinum* (Saxifragales: Grossulariaceae) (Figure 1b). Then, using a set of no‐choice and choice foraging assays, we showed that *R. bucephala* displays strong foraging preferences for *Ribes* plants compared maple *Acer* sp., a previously known hosts of this moth species. These findings have ecological implications to our understanding of the interactions between herbivorous insects and (previously undescribed) host plants, as well as the interactions between moth species, particularly in the Nordic region. Given that *P. bucephala* have been considered a transient pest in mainland Europe and the UK, our findings open questions in both applied and fundamental ecology.

## 2 MATERIAL AND METHODS

All data were analyzed in R software version 3.6.2 (R Development Team, 2010) while plots were made using the “ggplot2” package (Wickham, 2016).

### 2.1 Study organism: *Phalera bucephala*

The Buff‐tip moth *Phalera bucephala* Linnaeus (1758) is a nocturnal moth found in mainland Europe, the UK, and Asia, particularly Russia (Heath, 1983) (Figure 1a). *Phalera bucephala* are relatively large moths with reported wingspan of 55–68 mm (http://www.wildlifeinsight.com/buff‐tip‐moth‐phalera‐bucephala/). With its appearance of a “broken twig,” *P. bucephala* is unique in its appearance and readily recognized in moth traps. Importantly, this species has been considered a pest of apple trees in Lithuania during the times of the Soviet Union (Molis, 1970) as well as transient pests in the UK (Port & Thompson, 1980). *Phalera bucephala* outbreaks have been associated with increasing nitrogen content in the environment (Port & Thompson, 1980), and more recently, efforts to control *P. bucephala* have been published in the literature (Gninenko, 2009). Despite this, virtually no information about the natural history of this species is available in the literature, especially in regards to their dietary habits. This gap in our knowledge precludes us to understand the underlying ecological factors that can drive future outbreaks of this species and hamper our ability to predict how this transient pest will respond to ongoing climatic changes.

*Phalera bucephala* is a polyphagous species reportedly feeding on 10 host‐plant families (Table 1). Eggs are deposited in clusters (http://www.wildlifeinsight.com/buff‐tip‐moth‐phalera‐bucephala/), which have adaptive morphological structures in the egg to withstand potentially toxic substances exuding from host plants (Chauvin et al., 1974). As with other Lepidopterans, *Phalera bucephala* caterpillars are gregarious for the early stages of larval development but become solitary in the late larval instars (Sterling & Henwood, 2020). Larvae feeds in summer and autumn before pupating in September–October; pupation occurs in the soil and individuals overwinter as pupa (Sterling & Henwood, 2020). Although few physiological aspects of *P. bucephala* larval nutrition have been studied [e.g., food utilization (Evans, 1939) and lipid content (Schmidt & Osman, 1962)], *P. bucephala* remains a species with very scarce information of its nutritional ecology.

**TABLE 1:** Recorded dietary breadth *Phalera bucephala*. Data obtained from queries to the Natural History Museum in London (https://www.nhm.ac.uk)

Host‐plant family	Host‐plant species	Country
Aceraceae	*Acer platanoides*	Finland
	*Betula* sp.	Europe
Betulaceae	*Betula pendula*	Finland
	*Betula pubescens*	Finland
Corylaceae	*Corylus* sp.	Europe
	*Corylus* sp.	Europe
Leguminosae	*Laburnum* sp.	Europe
	*Robinia* sp.	Europe
Salicaceae	*Populus balsamifera*	Finland
	*Populus tremula*	Finland
Rosaceae	*Prunus* sp.	Europe
	*Rosa* sp.	Europe
	*Rosa rubrifolia*	Finland
Fagaceae	*Quercus* sp.	Europe
	*Quercus petraea*	British Isles
	*Quercus robur*	British Isles
	*Quercus robur*	Finland
Salicaceae	*Salix alba*	British Isles
	*Salix caprea*	Finland
	*Salix cinerea*	Finland
	*Salix lapponum*	Finland
	*Salix phylicifolia*	Finland
Tiliaceae	*Tilia* sp.	Europe
	*Tilia cordata*	Finland
	*Tilia platyphyllos*	Finland
Ulmaceae	*Ulmus* sp.	Europe
	*Ulmus* sp.	Finland
	*Ulmus procera*	British Isles
Caprifoliaceae	*Viburnum*	Europe

### 2.2 Observation site and specimen and food collections

*P. bucephala* caterpillars were observed in Ryd, a suburban area of the city of Linköping, Sweden (coordinates of the observation site: 58°24′29.6″N 15°34′08.7″E). Twenty‐eight caterpillars from the observation site were collected and placed in commercial buckets (20 L) containing c. 100 g of soil. Soil was collected with a spoon directly under the *Ribes alpinum* (Figure 1b) tree where caterpillars were observed. We also collected branches of the original plant *R. alpinum*, alongside with feeding caterpillars, using a scissor. Branches of c. 45 cm in length were pruned. This allowed us to collect caterpillars with minimum disturbance, while simultaneously collecting food (fresh leaves collected from the original plant). Water was provided by sprinkling tap water onto the leaves and soil in the bucket. Water was provided once a day in dried days or whenever showers occurred in the region. We allowed caterpillars to acclimatize to these conditions outdoors, with food and water ad libitum and fluctuating temperature and humidity similar to that of the environment, for 24 hr. We collected fresh branches of *R. alpinum* daily, both from the original plants and from a site within the adjacent forest (Rydskogen, Linköping, coordinates: 58°24′49.7″N 15°34′52.4″E). Caterpillars and host plants were identified using morphological traits, distribution information of species within families, public museum databases (e.g., Dyntaxa at www.dyntaxa.se) and, for caterpillars, a field guide (Sterling & Henwood, 2020); we also uploaded images to iNaturalist for identification (Nugent, 2018; Unger et al., 2020; Van Horn et al., 2018).

### 2.3 Field excursions

We had field excursions both to the surrounding observation site and to the adjacent forest. Excursions were made both on foot and on bicycles, and were made both early morning (between 7 and 9 a.m.) and evening (between 10 and 11 p.m.) for the subsequent 2 days following our observation or once a day (usually mornings) for the next 5 days. During mornings and evenings, *P. bucephala* feed on the edge of branches which facilitated our detection when walking pass or cycling pass, at a very slow speed, through *R. alpinum* trees. During these excursions, we followed paths (both in the suburban area and in the forest) and only covered the portion of the adjacent forest that was closest to the observation site. Nonetheless, sporadic more distant excursions through the forest and the neighborhood were also conducted at least twice a week since our first observation until the submission of the paper. We also collected morphometric data using a commercial ruler and a Denver Instrument^®^ scale with 0.001 g precision (Figure 1c).

### 2.4 Foraging behavior

**FIGURE 2:**
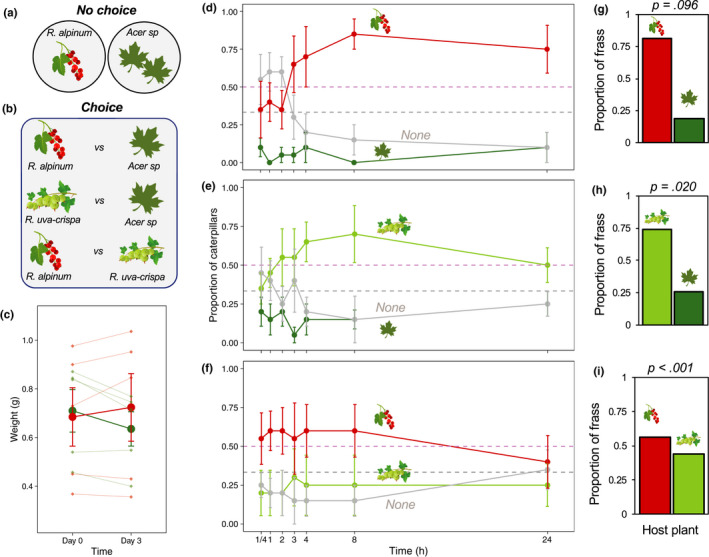
Empirical evidence reveals strong evidence of *Ribes* preference in *P. bucephala*. (a, b) Schematic example of the experimental design for the No‐choice (a) and Choice (b) assays. (c) *P. bucephala* weight (in g). (d, f) Proportion of cateprilars in each of the plants in the Choice experiments. (d) *Acer* sp. (maple) versus *R. alpinum* (mountain currant); (e) *Acer* sp. versus *R. uva‐crispa* (gooseberry); (f) *R. uva‐crispa* versus *R. alpinum*. “None” refers to the proportion of individuals foraging around the arena and not in any of the food plants. Red: *R. alpinum*, Light green: *R. uva‐crispa*; Dark green: *Acer* sp. (g–i) Proportion of frass in each of the quadrants containing the host plants in the Choice experiment. *p*‐Values obtained from chi‐square test with Monte‐Carlo simulations

Next, we wonder whether or not this population (a) could feed and grow in both currant (undescribed host) and maple (known host) and (b) there was foraging preference for currant as opposed to maple. To do this, we performed two sets of experiments: *No‐choice* and *Choice* experiments (Figure 2a,b).

#### 2.4.1 No‐choice foraging experiment

Ten caterpillars were weighed as described previously and allocated to either currant or maple dietary treatments (*N* = 5 per treatment), where they had food and water ad libitum for three consecutive days. To avoid introducing confounding effects of social treatment, each treatment was a feeding group of caterpillars since group feeding is common in this species. Within each group, we marked individuals with varying dot patterns in the upper portion of their heads (i.e., two dots on the left side, two dots on the right side, one dot in each of the sides in the same line, dots in diagonal with left side dot higher than right dot, and diagonal with right side dot higher than left dot). We weighted each individual at prior to the start of the experiment as well as after 3 days; the difference between the initial weight and the final weight after 3 days for each individual was used as a proxy for caterpillars' growth. Caterpillars were maintained outdoors throughout the duration of the experiment. We used two‐way ANOVA with time and diet treatment as factors for statistical inference.

#### 2.4.2 Choice foraging experiment

Five replicate groups containing four caterpillars were randomly assembled for the choice experiment. The choice experiment ran for three rounds in consecutive days, where the diet choices varied in each of the days (see below). No molts were observed, confirming that all experiments were ran in the third‐instar stage. Prior to the onset of every round, groups were starved for 30 min, before being released simultaneously in Styrofoam boxes with dimensions of c. 25 cm × 25 cm × 20 cm with food choices, a moist cotton wool at the bottom for moisture and water accessibility and covered with a lid to prevent caterpillars from escaping and to minimize visual cues during the experiment (see e.g., Morimoto, Nguyen, et al., 2019; Morimoto, Tabrizi, et al., 2019 for similar approach in other species). In the first round, groups were given a choice between mountain currant *R. alpinum* and maple *Acer* sp. We scored the number of caterpillars in each plant and the number of caterpillars that were in neither plant (i.e., “None”) (e.g., on the lid or on the sides of the box) at 15 min (1/4 hr), 1, 2, 3, 4, 8, and 24 hr after the onset of the experiment. Thus, in each round, caterpillars had three foraging possibilities (e.g., currant, maple, and no choice). In the second and third rounds, the experimental design was identical but with diet choices of gooseberry *R. uva‐crispa* or maple *Acer* sp., and gooseberry *R. uva‐crispa* or mountain currant *R. alpinum*, respectively (Figure 2b). Each round was analyzed separately. To analyze foraging decisions, we fitted a generalized additive model (GAM) and compared differences in the smooth parameter for these models between the three possible choices within each round. GAMs fitting matched the trends of the data, corroborating the goodness of fit of the model (see Figure S1). At the end of each round, we also drew a line which divided the box into halves, each representing a quadrant, and counted the number of frass present onto and around each of the food plants to calculate a proportion of time each caterpillar spent in each of the food quadrants. These data complemented our direct behavioral observations of the food choices. We calculated the proportion of frass in each of the food plants as the total number of frass onto and around the plant divided by the total number of frass in the box. We then compiled the data into a table and used chi‐square test for statistical inference with *p*‐values obtained with Monte‐Carlo simulations.

## 3 RESULTS

### 3.1 Undescribed host plant for *P. bucephala* caterpillars

We observed a population of >30 third instar *Phalera bucephala* caterpillars feeding on the apical portion of the stems of the new host plant—the mountain currant *Ribes alpinum* (Saxifragales: Grossulariaceae) (Figure 1a,b) in a suburban region of Linköping (Sweden). We collected 28 specimens (see Section 2) and found that caterpillars had average length of 4.210 ± 0.104 cm and average weight of 0.823 ± 0.057 grams (Figure 1c). Considering that a fully grown caterpillar can range from 6.5 to 7.5 cm (http://www.wildlifeinsight.com/6263/buff‐tip‐moth‐identification‐guide/), our data suggest that *R. alpinum* is a suitable host for *P. bucephala* growth and development. Other known host species were present in nearby regions, including the maple tree *Acer* sp., roses *Rosa* sp., *Betula* sp., oak *Quercus* sp., and *Viburnum* sp., all within 20–100 m of the observation site but none of which were used by *P. bucephala*. Field excursions and search in other mountain currant plants around the recorded region as well as in the adjacent forest (Rydskogen, Linköping) for the following week did not result in further encounters with the caterpillars, suggesting that the use of *R. alpinum* as host is yet relatively uncommon. When we offered *R. alpinum* fresh leaves collected from both the original plant and host plant from the aforementioned forest where gardening fertilizers were unlikely used, all 28 caterpillars (100%) were observed readily feeding on leaves from both sources, suggesting that *P. bucephala* caterpillars feeding on *R. alpinum* was unlikely a consequence of larval foraging preferences for increased nitrogen host plants in a particular location.

### 3.2 No‐choice experiment reveals potential costs of non‐*Ribes* feeding

Although not statistically significant (*Time × Host plant*: *F*_1,16_: 0.278, *p* = .605), there was a trend for caterpillars to decrease body weight after feeding for 3 days on maple *Acer* sp., whereas the opposite trend was found for caterpillars feeding on *Ribes alpinum* (Figure 2c). There were also no statistically significant main effects of time (*Time:* *F*_1,16_: 0.026, *p* = .872) or host plant on caterpillars' weight (*Host plant:* *F*_1,16_: 0.086, *p* = .772).

### 3.3 Choice experiment: *P. bucephala* caterpillars display strong preference for *Ribes* host plants

*P. bucephala* showed strong preference for *Ribes* plants in choice experiments (Figure 2d–f; see also Figure S1). For instance, when given a choice between *R. alpinum* and *Acer* sp., or *Ribes uva‐crispa* and *Acer* sp., caterpillars strongly preferred *Ribes* plants (*Contrast: R. alpinum* vs. *Acer:* 2.279 ± 0.371; *p* < .001; *R. uva‐crispa* vs. *Acer:* 1.175 ± 0.238; *p* < .001; Table S1). Interestingly, for both rounds of choice with *R. alpinum* and *R. uva‐crispa*, caterpillars seemed to forage for the first few hours after the onset of the experiment, shown by the relatively higher proportion of caterpillars in neither of the plants. However, after approximately 2 hr, caterpillars displayed a sharp preference for *Ribes* plants that were sustained for the remaining of the experiment (Figure 2d,e), although this was more strongly observed for *R. alpinum*. This pattern emerged as a result of foraging caterpillars choosing to feed on *Ribes* plants, which generated almost mirror images between the sigmoidal curves of the proportion of caterpillars in *Ribes* plants and the proportion of caterpillars foraging in the arena (Figure 2d,e). The proportion of caterpillars feeding on *Acer* sp. remained low and linear throughout the experiments (Figure 2d,e). In fact, the sigmoidal pattern of diet choice observed for *R. alpinum* was significantly different from the linear pattern observed for *Acer* sp. (edf = 1.899, Chisq = 6.864; *p* = .043; Table S1), although similar, the sigmoidal pattern of foraging preference for *R. uva‐crispa* was not statistically different from the linear pattern observed for *Acer* sp. (edf = 1.657, Chisq = 1.619; *p* = .453; Table S1). Together, these results revealed that *P. bucephala* displayed strong preference for *R. alpinum* and, to a smaller extent, *R. uva‐crispa* (Figure 2d,e). To confirm that *P. bucephala* had stronger preference for *R. alpinum* as opposed to *R. uva‐crispa*, we ran the final round of the foraging choice experiment with both plants as food options. Interestingly, the sigmoidal patterns observed in the choice rounds with *Acer* sp. disappeared (Figure 2f). Yet, *P. bucephala* still displayed stronger preference for *R. alpinum* (*Gooseberry* vs. *Currant:* 0.853 ± 0.208; *p* < .001; Table S1) although no nonlinear trends were observed in the data (Figure 2f; Table S1). This preference was nevertheless weaker, and 24 hr after the onset of the choice experiment, the proportion of caterpillars in *R. alpinum*, *R. uva‐crispa* and foraging (*none*) was equal and equivalent to a random distribution across the three options (Figure 2f). The proportion of frass in each of the food choices corroborated these results (see Figure 2g–i).

## 4 DISCUSSION

In this study, we described for the first time a previously unknown host plant for *P. bucephala*, a moth species that has been considered a transient pest in Europe and the UK. This is the first report of a Notodontid moth feeding on Grossulariaceae host plants, which expands our understanding of the family‐level diet breadth of Notodontid moths. With dietary no‐choice and choice experiments, we showed that *P. bucephala* displayed strong dietary preferences for *Ribes* plants, particularly *R. alpinum*, revealing some level of dietary specialization to this undescribed host. These results have implications to both the natural history and ecology of Notodontid moths, as well as to the interaction of *P. bucephala* with other moth species. Below, we discuss our findings and highlighting their ecological significance.

### 4.1 Strong preference for previously undescribed host

Understanding larval foraging decisions in complex heterogenous dietary habitats has been an important topic of research in evolutionary ecology (Schultz, 1983; Singer & Stireman, 2001). In many circumstances, larvae (especially caterpillars) can display accurate foraging decisions and given the availability of more suitable hosts nearby, change hosts to match host quality with larval preferences (Schultz, 1983) (see also evidence for accurate choice in other insect larvae; e.g., beetles: Messina, 1982, flies: Morimoto, Tabrizi, et al., 2019). In this study, we showed that, although feeding in a previously undescribed host, *P. bucephala* caterpillars showed strong preferences for *Ribes* plants over a previously known host for this species. This preference was particularly higher for *Ribes alpinum*, the plant in which our original natural history observation was made. Given that butterflies acquire preferences for novel hosts during early exposure to novel hosts' odors and transmit these acquired preferences to their offspring (Gowri et al., 2019), our findings suggest that the association between *P. bucephala* and *Ribes* host plants could to persist over generations.

### 4.2 Could *Ribes* host support range expansion?

Novel host‐plant associations are crucial for distribution range of many insects and particularly relevant in studies of invasion of insect pests (Bertheau et al., 2010). Chemical similarity between novel and ancestral plants affects the ability of insects' to utilize the novel host and also alters insect population dynamics in ways that can facilitate range expansion (Ammunét et al., 2011). For instance, chemical similarity between two pine trees (i.e., the ancestral host *Pinus contorta* var. *latifolia* and the novel host *Pinus banksiana*) likely underpinned the successful expansion of the mountain pine beetle (*Dendroctonus ponderosae*) to jack pine forests (Erbilgin et al., 2014). Here, we showed that *P. bucephala* can feed in *Ribes*, a host which belongs to a previously unknown family of plants that can be used by this species. We do not know whether our observation is evidence for preference to an evolutionarily novel Grossulariaceae host or if Notodontid moths (in particular, *P. bucephala*) has evolved feeding abilities to Grossulariaceae plants in the past but has only been documented now. Future phylogeographic studies should provide insights into this. However, if our observation does represent evidence for the evolution of feeding into a novel host, this could open up new avenues through which *P. bucephala* could expand its range, either by shifting to hosts with similar chemical composition to *Ribes* or by using *Ribes* as hosts in novel habitats. For instance, while *P. bucephala* is relatively common in Europe, there have not yet been records, to our knowledge, of this species in North America (Figure 1). Yet, *R. alpinum* (and other *Ribes* species such as e.g., Gooseberry *Ribes uva‐crispa*) are widely distributed in the Northern hemisphere. If *P. bucephala* can use *Ribes* species as host, it is at least possible that *P. bucephala* could expand its distribution range (naturally or by human introduction) to North America. Moreover, even if *P. bucephala* does not reach North America, it is possible that climate change, which imposes particularly strong effects in high latitudes (e.g., Bunn et al., 2007), could contribute to an increase in temperature that leads to an expansion in latitudinal range of *P. bucephala* supported by the use of *Ribes* sp. as hosts in the south of Europe.

### 4.3 Species interactions: can *P. bucephala* outcompete *Ribes*‐specialists?

**FIGURE 3:**
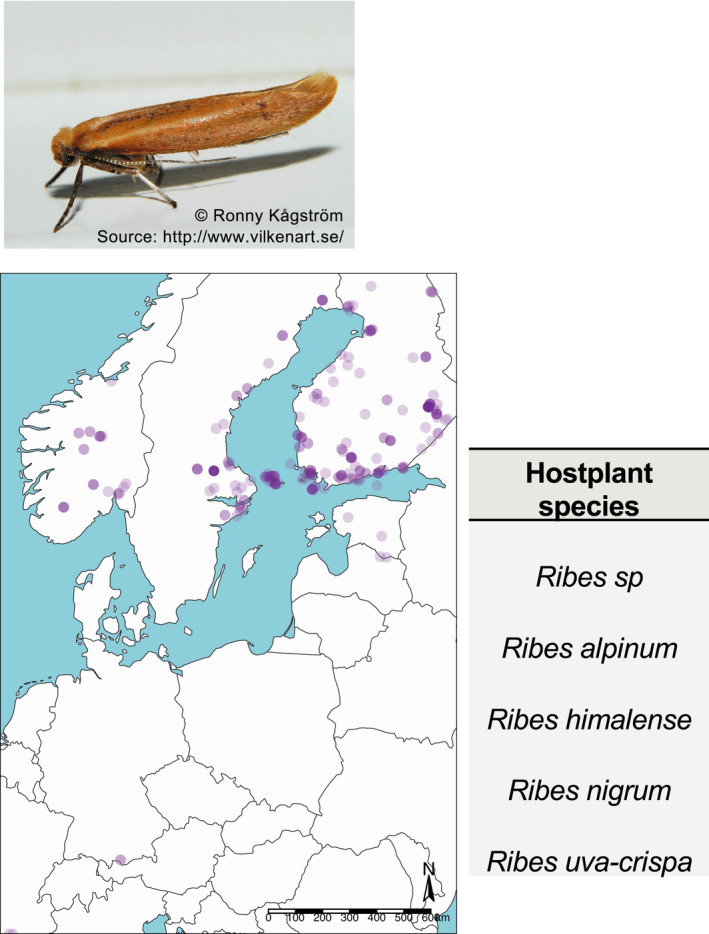
Potential for interspecific competition with *Ribes*‐specialist moths in the Nordic region. *Ribes*‐specialist moth of the Nordic region *Euhyponomeutoides ribesiella*. *Top panel*: *E. ribesiella* specimen (image from the public domain). *Right panel*: *E. ribesiella* recorded diet breadth. Note that *E. ribesiella* feeds on *Ribes* species. *Main panel*: All GBIF recorded observations (*N* = 280) of *E. ribesiella* across its distribution range. Data queried from the GBIF database on the July 27, 2020

Although *P. bucephala* is unlikely to be under threat of extinction, our observation that *P. bucephala* uses *R. alpinum* as hosts raises many questions with important implications to interspecific interaction in the Nordic region. For instance, the moth *Euhyponomeutoides ribesiella* de Joannis (1900) (Lepidoptera: Yponomeutidae) is known to be a *Ribes‐*specialist moth of the Nordic region (Figure 3). It is possible that *E. ribesiella* could face interspecific competition with *P. bucephala* if the prevalence of *Ribes* feeding in the latter species increases. As *P. bucephala* is a polyphagous species, this competition could displace the specialist *E. ribesiella* thereby decreasing the available habitats in which *E. ribesiella* can utilize. In turn, this could decrease *E. ribesiella* population sizes and population connectivity (i.e., fragmentation), ultimately leading to *E. ribesiella* extinction. Better understanding such interspecific competition between a generalist and a specialist species in high latitudes could shed light into the worldwide pattern of functional homogenization observed across taxa (Clavel et al., 2011), including herbivorous insects (Deguines et al., 2016; Harvey & MacDougall, 2015; Merckx & Van Dyck, 2019). A key question for future research is as follows: what are the implications of *P. bucephala* feeding no Grossulariaceae to other herbivorous insect species?

## 5 CONCLUSION

We observed, for the first time, *P. bucephala* feeding on *Ribes*. Such dietary flexibility to host plant families suggest that *P. bucephala* and possibly all Notodontids possess strong physiological robustness to cope with varying phytochemical defences (see e.g., Volf et al., 2015, review by Ali & Agrawal, 2012). This opens up the potential for *P. bucephala* to become a good study system to test dietary shifts, plastic responses to different diets, as well as physiological mechanisms used by herbivorous insects to cope with host‐plant defences. Future studies should investigate the phylogeographic patterns of Notodontid moths and Grossulariaceae hosts to better understand the origins of this relationship, as well as the broader ecological implications of generalists acquiring the potential to exploit novel host plants, particularly those used by other specialist species. This can help us better understand the origin and consequences of ecological dynamics driven by diet breadth, helping raise exciting new questions (and answers) for basic, applied and conservation ecology of insects.

## CONFLICT OF INTEREST

The authors have no conflict of interests to declare.

## AUTHOR CONTRIBUTIONS

**Juliano Morimoto:** Conceptualization (lead); Data curation (lead); Formal analysis (lead); Investigation (equal); Methodology (lead); Visualization (lead); Writing‐original draft (equal); Writing‐review & editing (lead). **Zuzanna Pietras:** Conceptualization (supporting); Formal analysis (supporting); Investigation (equal); Writing‐original draft (equal); Writing‐review & editing (supporting).

## Supporting information

Supplementary Material

## Data Availability

Raw data are available in Dryad https://doi.org/10.5061/dryad.pk0p2ngkx.
