# Supplementary material for: Strong foraging preferences for Ribes alpinum (Saxifragales: Grossulariaceae) in the polyphagous caterpillars of Buff‐tip moth Phalera bucephala (Lepidoptera: Notodontidae)
Source: Ecol Evol. 2020 Nov 11;10(24):13583–92. doi: 10.1002/ece3.6981 (PMC7771136; doi:10.1002/ece3.6981)
Supplement: Supplementary file 1 — Supplementary Material [file ECE3-10-13583-s001.pdf]

Supplementary Material: "Strong foraging preferences for *Ribes alpinum* (Saxifragales: Grossulariaceae) in the polyphagous caterpillars of Buff tip moth *Phalera bucephala* (Lepidoptera: Notodontidae)"

Authors: Juliano Morimoto[1,*], Zuzanna Pietras[2]

Authors' Affiliations:

1 School of Biological Sciences, University of Aberdeen, Zoology Building, Tillydrone Ave, Aberdeen AB24 2TZ

2 Department of Physics, Chemistry and Biology (IFM), Linköping University, Sweden

*Correspondence: juliano.morimoto@abdn.ac.uk

Authors' contributions. JM and ZP observed and collected the specimens and the body size information. JM analysed and plotted the data. Both authors contributed to the writing of the report.

Table S1. Complete output of GAM models for in the Choice experiment. Note that the reference level for comparison is the first food plant (e.g., *Acer* in the *Acer vs R. alpinum)*.

Acer vs R. alpinum				
Parametric	***coefficients:***			
	**Estimate**	**std error**	**z-value**	**p-value**
(Intercept)	-1.4759	0.3536	-4.174	**<0.001**
R. alpinum	2.2791	0.3717	6.132	**<0.001**
None	1.6617	0.3944	4.214	**<0.001**
Approximate	***significance***	***of***	***smooth***	***terms:***
	edf	Ref.df	Chi.sq	p-value
s(R. alpinum)	1.899	2.176	6.864	**0.043**
s(None)	1.675	1.991	9.9	**0.005**
R-sq.(adj) =	*0.48*	*Explained deviance:*	*46.40%*	
Acer vs R. uva-crispa				
Parametric	***coefficients:***			
	**Estimate**	**std error**	**z-value**	**p-value**
(Intercept)	-0.419	0.208	-2.014	**0.044**
R. uva-crispa	1.1757	0.2386	4.928	**<0.001**
None	0.575	0.261	2.202	**0.0270**
Approximate	***significance***	***of***	***smooth***	***terms:***
	edf	Ref.df	Chi.sq	p-value
s(R. uva-crispa)	1.657	1.954	1.619	0.453
s(None)	1.674	1.98	2.452	0.254
R-sq.(adj) =	*0.263*	*Explained deviance:*	*24.50%*	
R. uva-crispa vs R. alpinum				
Parametric	***coefficients:***			
	**Estimate**	**std error**	**z-value**	**p-value**
(Intercept)	-0.05884	0.17408	-0.338	0.735
R. alpinum	0.85391	0.20808	4.104	**<0.001**
None	-0.1505	0.25715	-0.585	0.558
Approximate	***significance***	***of***	***smooth***	***terms:***
	edf	Ref.df	Chi.sq	p-value
s(R. alpinum)	1	1	0.854	0.356
s(None)	1.059	1.115	1.369	0.243
R-sq.(adj) =	*0.191*	*Explained deviance:*	*17.80%*	

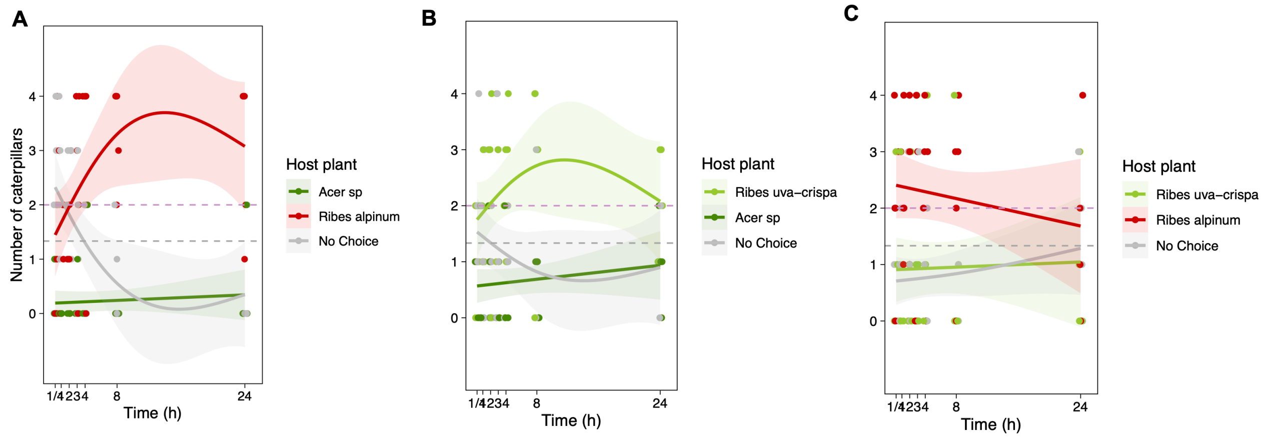

Figure S1. (*a* - *c*) GAM smoothed curves for the Choice experiment analysis (compare with Fig 2d-f).
